# Porin 1 Modulates Autophagy in Yeast

**DOI:** 10.3390/cells10092416

**Published:** 2021-09-14

**Authors:** Filomena Broeskamp, Elizabeth S. M. Edrich, Oskar Knittelfelder, Lisa Neuhaus, Thorsten Meyer, Jonas Heyden, Lukas Habernig, Florian Kreppel, Campbell W. Gourlay, Patrick Rockenfeller

**Affiliations:** 1 Chair of Biochemistry and Molecular Medicine, Center for Biomedical Education and Research (ZBAF), University of Witten/Herdecke (UW/H), Stockumer Str. 10, 58453 Witten, Germany; filomena.broeskamp@su.se (F.B.); Lisa.neuhaus@uni-wh.de (L.N.); thorsten.meyer@uni-wh.de (T.M.); jonas.heyden@uni-wh.de (J.H.); Florian.kreppel@uni-wh.de (F.K.); 2 Department of Molecular Biosciences, The Wenner-Gren Institute, Stockholm University, 10691 Stockholm, Sweden; Lukas.habernig@su.se; 3 Kent Fungal Group, School of Biosciences, University of Kent, Canterbury CT2 7NJ, UK; esme2@kent.ac.uk; 4 Max Planck Institute of Molecular Cell Biology and Genetics, 01307 Dresden, Germany; oskar.knittelfelder@gmail.com; 5 Institute of Molecular Biosciences, NAWI Graz, University of Graz, 8010 Graz, Austria

**Keywords:** autophagy, voltage dependent anion channel (VDAC), phosphatidylethanolamine, phosphatidylserine decarboxylase

## Abstract

Autophagy is a cellular recycling program which efficiently reduces the cellular burden of ageing. Autophagy is characterised by nucleation of isolation membranes, which grow in size and further expand to form autophagosomes, engulfing cellular material to be degraded by fusion with lysosomes (vacuole in yeast). Autophagosomal membranes do not bud from a single cell organelle, but are generated de novo. Several lipid sources for autophagosomal membranes have been identified, but the whole process of their generation is complex and not entirely understood. In this study, we investigated how the mitochondrial outer membrane protein porin 1 (Por1), the yeast orthologue of mammalian voltage-dependent anion channel (VDAC), affects autophagy in yeast. We show that *POR1* deficiency reduces the autophagic capacity and leads to changes in vacuole and lipid homeostasis. We further investigated whether limited phosphatidylethanolamine (PE) availability in *por1*∆ was causative for reduced autophagy by overexpression of the PE-generating phosphatidylserine decarboxylase 1 (Psd1). Altogether, our results show that *POR1* deficiency is associated with reduced autophagy, which can be circumvented by additional *PSD1* overexpression. This suggests a role for Por1 in Psd1-mediated autophagy regulation.

## 1. Introduction

Macroautophagy, hereafter referred to as autophagy, represents a cellular recycling mechanism, which regulates degradation of misfolded, oxidised, aggregated, or simply unneeded proteins. Furthermore, autophagy can facilitate the degradation of entire organelles, such as mitochondria or peroxisomes [1]. Autophagy thus represents a major cellular means to maintain homeostasis and protect from age-related damage [2]. During autophagy, an isolation membrane is nucleated and extended to form an autophagosome, which is used to enclose the material to be degraded. The lipid material making up the autophagosomal membrane does not simply derive from a single organelle, but uses multiple lipid sources, such as the ER [3], mitochondria [4], Golgi [5,6], plasma membrane [7,8], endosomes [9,10], and lipid droplets [11,12]. However, it has recently been suggested that the extension of the autophagosomal membrane occurs de novo at ER–autophagosome contacts. This process involves Faa1-mediated channelling of activated fatty acids into phospholipids, which are used in autophagosomal membrane expansion [13]. The availability of fatty acids [14,15,16] and phosphatidylethanolamine (PE) [17] has further been shown to be a limiting factor for autophagy.

Phospholipid synthesis occurs predominantly at the ER and, to some extent, within mitochondria. Mitochondria can contribute to PE synthesis, which is used widely within the cell, as well as synthesis of those lipids specific to the organelle, such as cardiolipin (CL) and phosphatidylglycerol (PG). Phosphatidic acid (PA) represents the precursor for CL and PG synthesis [18], whereas PE is generated in the mitochondrion through decarboxylation of phosphatidylserine (PS) by the PS decarboxylase Psd1 [19,20]. In yeast, Psd1 was thought to be specific to mitochondria [20]; however, recent evidence suggests it can also localise to the ER, where it promotes PE synthesis upon starvation [21]. Three alternative pathways have been described for PE production in yeast, Psd2-mediated PS decarboxylation, the CDP–ethanolamine branch of the Kennedy pathway, and lyso-PE reacylation by Ale1 [22]. In yeast, mitochondrial PE production via Psd1 represents the major route [22]. This route relies on the delivery of PS as a PE precursor from the ER to mitochondria. Efficient PS transfer from the outer to the inner mitochondrial membrane is carried out by the lipid transfer complex Ups2–Mdm35 [18,23]. Alternatively, Psd1 can process PS in the outer mitochondrial membrane in trans at the MICOS contacts, the site where outer and inner mitochondrial membranes come into close contact [18,24]. The CL precursor PA is translocated from the outer to the inner mitochondrial membrane in a similar way to Ups2–Mdm35-dependent PS translocation using Ups1–Mdm35 [18,25].

These lipid transport systems and the communication between the ER and mitochondria might represent important factors not only for co-ordinating lipid metabolism, but also in regulating autophagy. Such communication is facilitated by proteins or protein complexes, which bring the two organelles into close contact. These include membrane contact sites (MCS) between the ER and mitochondria, in yeast, namely ERMES [26,27] (ER–mitochondria encountering structure) and EMC (ER–mitochondria complex) [28].

In this study, we investigate the possibility that VDAC may also play a role in lipid transport and the process of autophagy, which may suggest a role in mitochondria–ER contacts. VDACs/porins are mitochondrial outer membrane proteins, which allow for metabolite exchange between the mitochondrial intermembrane space and the cytosol. They, thus, act as controllers of metabolic flux into the mitochondria [29]. Human VDAC1 consists of a β-barrel containing 19 amphipathic β-strands and an N-terminus formed of α-helical segments, which wedge into the channel and partially block it [30]. Mammalian VDACs have been described as components of ER–mitochondria tethering complexes regulating Ca^2+^ homeostasis [31]. Whether the yeast VDACs Por1 or Por2 have such tethering functions is unexplored, but large-scale Por1 interaction data suggest physical and/or genetic interactions with the ERMES components Mdm10, Mdm12, Mdm34, and Mmm1 [27,32,33,34,35]. Por1 is the most abundant protein in the yeast outer mitochondrial membrane, with up to 16,000 copies per mitochondrion [36]. It, thus, represents a very suitable docking scaffold for proteins mediating tethering to mitochondria and not only to mediate Ca^2+^ transfer, but also to regulate lipid exchange.

In this study, we examined how Por1 affects autophagy and lipid metabolism in yeast. Our results reveal that the expression level of Por1 is critical for proliferation, lipid homeostasis, and autophagy. We suggest that Por1 harbours essential functions in the co-ordination of lipid synthesis and inter-organelle communication, which impact the regulation of autophagy.

## 2. Materials and Methods

### 2.1. Yeast Strains and Growth Conditions

All experiments were carried out in BY4741 (*Mat***a** *his3*Δ1; *leu2*Δ0; *met15*Δ0; *ura3*Δ0) obtained from Euroscarf. The single-deletion strain of *POR1* was obtained from the Euroscarf knock-out collection. Yeast strains carrying *pATG8–EGFP–ATG8* fusions were generated according to the method by Janke et al. [37], using a modified pYM-N37 plasmid (pYMpATG8) as a template and primers as described in Eisenberg et al. [38]. Yeast strains carrying chromosomal *VPH1*–yEGFP (introduced with a *URA3* selection marker) were a kind gift from Sabrina Buettner. For expression of Por1, we used PCR amplification of *POR1* using the following primers:

*POR1*-fwd: atcaCGGCCGATGTCTCCTCCAGTTTACAG;

*POR1*-rev: atcaGAGCTCTCAAGCGTCGAAGGACAAAG.

**Figure 1:**
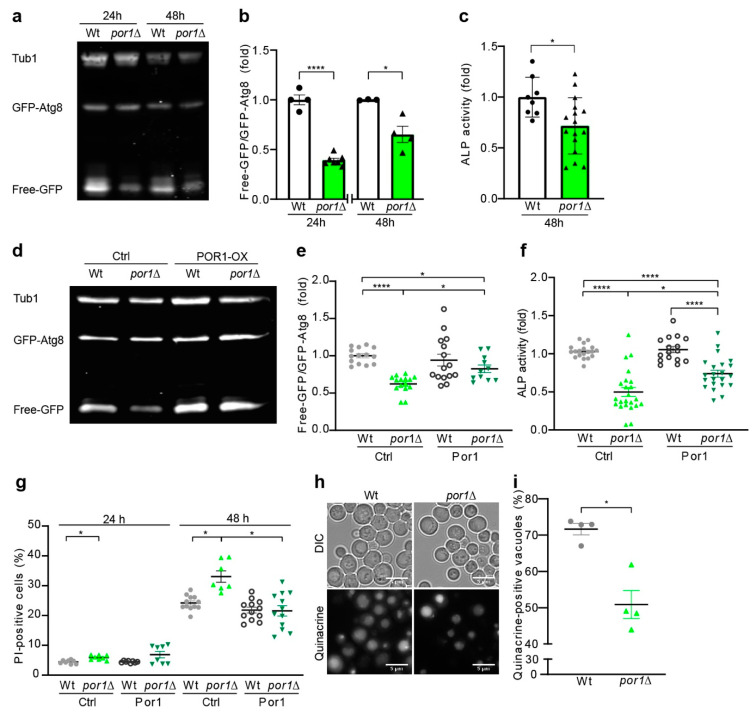
Deletion of *POR1* impaired autophagy. (**a**–**c**) The autophagic flux was reduced in *por1*∆ at 24 h and 48 h (growth on YPD medium). GFP liberation using GFP–Atg8-expressing cells was detected by immunoblotting (**a**) and densitometric quantification of free-GFP/GFP–Atg8 ratios (**b**). The immunoblots were probed with GFP- and tubulin-specific antibodies, and the signals were normalised to wildtype (Wt) (*n* ≥ 3). Alkaline phosphatase activity was assessed using the Pho8∆60 assay indicative for autophagic flux at 48 h normalised to wildtype (*n* ≥ 8) (**c**). (**d**–**f**) Reduced autophagic flux in *por1*Δ can be restored by *POR1* expression after 24 h of induction on SCD/SCG (1:1). A representative immunoblot of wildtype and *por1*Δ cells with and without expression of *POR1* is shown in (**d**), and densitometric quantification of free-GFP/GFP–Atg8 ratios is depicted in (**e**). Immunoblots were probed with GFP- and tubulin-specific antibodies and signals were normalised to wildtype levels (*n* ≥ 11). Alkaline phosphatase activity was assessed at 24 h using the Pho8∆60 assay (*n* ≥ 18) (**f**). (**g**) Chromosomal *POR1* deletion triggers cell death, which can be rescued by *POR1* expression from a plasmid and induction on SCD/SCG (1:1). Cell death was assessed by PI staining at indicated time points and quantified using flow cytometry (*n* ≥ 7). (**h**,**i**) Quinacrine staining at 24 h on SCD/SCG (1:1) media monitors reduced vacuole acidification in *por1*Δ cells. Representative micrographs are shown in (**h**), and cells with acidic vacuoles are quantified in (**i**) (*n* = 4). Statistical analysis was performed with Graphpad Prism. *p* values indicate statistical significance of the Student *t*-test in (**b**,**c**,**i**) and Welch’s ANOVA in (**e**,**f**,**g**) with * *p* < 0.05 and **** *p* < 0.0001.

A restriction digest of the PCR product was carried out with EagI (CGGCCG) and SacI (GAGCTC), which was then ligated into the similarly digested pESC-HIS plasmid (Stratagene). *PSD1* was also expressed from a pESC-HIS plasmid, the construction of which was previously described [17]. Transformation of plasmids into yeast cells was performed using the lithium acetate method [39]. At least three different clones were tested after plasmid transformation or genomic replacement to rule out clonogenic variations. All experiments were carried out in synthetic complete (SC) medium, except for the experiments shown in Figure 1a–c and Figure S1a,b, which were carried out on yeast peptone (YP) medium. YP medium contains 1% yeast extract (BD), 2% peptone (BD), and 2% glucose as a carbon source for YPD medium or 3% glycerine for YPGly medium, respectively. SC medium contains 0.17% yeast nitrogen base (Difco), 0.5% (NH_4_)_2_SO_4_, and 30 mg/L of all amino acids (except 80 mg/L histidine and 200 mg/L leucine), 30 mg/L adenine, and 320 mg/L uracil with 2% glucose. All media were prepared with ultrapure water (MilliQ) and subsequently autoclaved (20 min, 121 °C, 110 kPa). Amino acid mixtures were sterilised separately as 10× stocks and added after autoclaving; this also applies to glucose and galactose stocks. All yeast cultures were inoculated from a stationary overnight culture to an OD_600_ = 0.1 or 0.15, and then grown at 30 °C and 145 rpm shaking for indicated time points. For Gal10-driven expression of Por1 or Psd1, we used SC medium with 1% D-glucose and 1% D-galactose (1:1 SCD/SCG) or, in the case of Psd1 expression, SC medium with 1.5% D-galactose and 0.5% D-glucose. These mixed-carbon source media were used to optimise growth conditions for *por1*∆, which has severe growth defects on galactose-only-containing growth media, while, at the same time, allowing for expression of the constructs. Cells were shifted to SCD/SCG medium 5 h after inoculation. Aliquots were harvested at indicated time points to study survival, vacuolar morphology or autophagy.

All autophagy induction experiments were carried out on SC medium. For rapamycin-induced autophagy, rapamycin (dissolved in DMSO) was supplemented to a final concentration of 30 nM at 6 h after inoculation. Autophagy induction by nitrogen starvation was similarly performed as described before [40]. In brief, cells were inoculated in 2% SCD and shifted to nitrogen starvation medium consisting of SC medium without (NH_4_)_2_SO_4_ and amino acids after 8 h of growth, with two steps of washing with ultrapure water. Samples were adjusted to an OD_600_ of 1.

### 2.2. Growth Assays

Wildtype, *por1*∆, and *POR1-* or *PSD1-*overexpression strains were inoculated from overnight cultures to an OD_600_ of 0.1 (1 mL total volume per well) in 24-well plates (Sarstedt) in two or three independent experiments, each containing at least three biological replicates. The plate was automatically measured for 24–48 h using a BMG LabTech SPECTROstar^Nano^ plate reader with double orbital shaking at 400 rpm and 30 °C, with OD_600_ measurements every 30 min. Growth curves were plotted in GraphPad Prism.

### 2.3. Yeast Autophagy Measurements

Autophagy was measured by monitoring the cytosol-to-vacuole translocation of Atg8 using fluorescence microscopy and immunoblotting (GFP liberation assay) of cells/cell extracts from strains carrying a GFP–Atg8 fusion protein expressed under its endogenous promoter and at its natural chromosomal locus [41,42].

Quantification of micrographs was performed from blinded pictures from 3 clones per replicate. Autophagic cells were defined as cells exhibiting clear vacuolar GFP fluorescence and expressed as the fraction of viable (PI-negative) cells. All micrographs were taken after 24 h, either on SCD/SCG (1:1) media or nitrogen starvation media. The quantification on SCD/SCG media contains 11 micrographs per strain, with 96–525 cells per micrograph; in total, 2871–4054 cells were evaluated. The quantification of GFP-positive vacuoles on nitrogen starvation includes 4 micrographs per replicate (in total 12) per strain. In total, 1300–1550 cells were manually counted per genotype.

For Western blot analysis, cell equivalents of an OD_600_ of 3 were harvested at indicated time points, and cell extracts were obtained from chemical lysis as described in [43]. Proteins were collected by centrifugation and resuspended in 75 µL 1× loading buffer (125 mM Tris-HCl, adjusted to pH 6.8; 20% glycerol; 3% SDS; 2% DTT; 0.1% bromophenol blue), and heated to 95 °C for 10 min. Samples were centrifuged at 13,000 rpm for 12 s and 10 µL or 15 µL of the supernatant was used for standard SDS-PAGE. Immunoblotting followed standard procedures, with transfer of proteins to a 0.45 µm nitrocellulose membrane and probing with antibodies against GFP (Roche, #11814460001, 1:5000, Basel, Switzerland), tubulin (Abcam, ab184970, 1:20,000, Cambridge, MA, USA), glyceraldehyde-3-phosphate dehydrogenase (GAPDH) (Life Technologies, MA515738, 1:5000, Carlsbad, CA, USA), Psd1 (gift from G Daum), or VDAC/porin (Abcam, ab110326, 1:5000) [42]. As secondary antibodies, we used IRDye goat anti-mouse (Licor, 926–68,070, 1:20,000, Lincoln, NE, USA) or IRDye goat anti-rabbit (Licor, 92840028, 1:20,000). Signals were recorded with Odyssey Glx, with automatically determined exposure times. For quantification of immunoblotting signals, a rectangular volume tool of Image Studio (Licor) with background correction set to “local background” was used. To compare different blots, each blot was normalised to at least two control samples before data was pooled for further analysis.

Additional biochemical autophagy measurements were performed using the quantitative Pho8∆60 assay (also called ALP assay) for nonspecific autophagy, essentially as described earlier [44]. In brief, OD_600_ = 8 equivalents of stationary yeast cell cultures were harvested and disrupted in 1.5 mL Eppendorf tubes with 100 µL of acid-washed glass beads using a Retsch mill Tissue Lyser with agitation for 3 min at 25 Hz. Subsequently, the protein concentration was measured applying a Bio-Rad protein assay. A total of 1.5 µg of total protein was used for the assay. The reaction was started by adding 50 µL α-naphthyl phosphate, and then incubated at 30 °C for 20 min. The reaction was stopped by adding 200 µL glycine–NaOH and fluorescence was measured using Tecan Infinite M Plex. To correct for intrinsic (background) ALP activity, the ALP activity of corresponding strains without Pho8∆60 manipulation was assessed and subtracted.

### 2.4. Analysis of Cell Death

Propidium iodide (PI) staining was used to determine loss of membrane integrity. Cells were harvested in 96-well plates at indicated time points and resuspended in 250 µL of 100 µg/L PI in PBS, and incubated in the dark for 10 min at room temperature. After incubation, cells were washed once with 250 µL PBS and analysed via flow cytometry (Beckmann Coulter Cytoflex). A total of 30,000 cells per strain and condition were measured and analysed with CytExpert software.

### 2.5. Assessment of Vacuole Morphology and Function

Vacuole morphology was determined using an endogenously GFP-tagged Vph1 strain. *S. cerevisiae* strains (wildtype and *por1*∆) were inoculated to an OD_600_ = 0.1 from overnight cultures in SCD medium and shifted to SCD/SCG (1:1) medium after 5 h. OD_600_ equivalents of 1.0 were harvested at 24 h after shift and mounted on microscopy slides. Fluorescence was detected using a Nikon Eclipse Ni-U fluorescence microscope with a Hamamatsu Orca-Spark C11440-36U monochromatic camera and Nikon Intensilight C-HGFI illumination system. Images were captured, saved, and processed using NIS-Elements BR 4.13.05 64-bit and ImageJ software. For quantification, 416–687 cells per genotype and condition were manually counted.

Functional vacuole acidification was assessed using the fluorescent dye quinacrine. Vacuolar quinacrine internalisation depends on vacuolar acidification. Hence, quinacrine fluorescence inside the vacuole can be used as a measure of vacuole function [45,46]. Wildtype and *por1*∆ cells were grown in the same manner as described for Vph1–GFP microscopy. After 24 h, cells were washed with 500 μL of YEPD containing 100 mM HEPES (pH 7.6). After centrifugation, the pellet was resuspended in 500 μL of YEPD with 100 mM HEPES (pH 7.6) and 200 μM quinacrine, and incubated for 10 min at 30 °C and 145 rpm. Afterwards, samples were transferred to ice and incubated for 5 min. After centrifugation, cells were washed twice in 500 μL ice-cold HEPES buffer supplemented with 2% D-glucose. The cells were pelleted by centrifugation, and then analysed using a Nikon Eclipse Ni-U epifluorescence microscope. For quantification, 1600–1930 cells per genotype were manually counted.

### 2.6. Lipid Extraction and Quantification by Shotgun Mass Spectrometry

Yeast cultures were inoculated from stationary overnight cultures in SCD to fresh SCD medium to an OD_600_ of 0.1, and shifted to SCD/SCG (1:1) media after 5 h. In total, 2 OD_600_ units were harvested after 24 h and homogenised with 0.5 mm zirconia beads in a cooled tissue lyser for 2 × 10 min at 30 Hz in 300 µL IPA. The whole homogenate was evaporated in a vacuum desiccator to complete dryness. Lipid extraction was performed according to [47,48,49]. In brief, 700 µL internal standard mix in 10:3 MTBE/MeOH was added to each sample and vortexed for 1 h at 4 °C. After the addition of 140 µL H_2_O, samples were vortexed for another 15 min. Phase separation was induced by centrifugation at 13,400 rpm for 15 min. The organic phase was transferred to a glass vial and evaporated. Samples were reconstituted in 300 µL 1:2 MeOH/CHCl_3_. For lipidome, 5 µL of sample were diluted with 95 µL 4:2:1 IPA/MeOH/CHCl3 + 7.5 mM ammonium formate.

Mass spectrometric analysis was performed on a Q Exactive instrument (Thermo Fisher Scientific, Bremen, DE, USA) equipped with a robotic nanoflow ion source TriVersa NanoMate (Advion BioSciences, Ithaca, NY, USA) using nanoelectrospray chips with a diameter of 4.1 µm. The ion source was controlled by the Chipsoft 8.3.1 software (Advion BioSciences). Ionisation voltage was +0.96 kV in the positive and −0.96 kV in the negative mode; back pressure was set at 1.25 psi in both modes. Samples were analysed by polarity switching [49]. The temperature of the ion transfer capillary was 200 °C; S-lens RF level was set to 50%. Each sample was analysed for 18 min. FT-MS spectra were acquired within the range of *m/z* 400–1000 from 0 min to 0.2 min in the positive mode, and within the range of *m/z* 350–1200 from 6.2 min to 6.4 min in the negative mode at a mass resolution of R *m/z* 200 = 140,000, automated gain control (AGC) of 3 × 10^6^, and with a maximal injection time of 3000 ms. Ergosterol was determined by parallel reaction monitoring (PRM) FT-MS/MS between 0.2 and 1.7 min. For FT-MS/MS, micro-scans were set to 1, isolation window to 0.8 Da, normalised collision energy to 12.5%, AGC to 5 × 10^4^, and maximum injection time to 3000 ms. t-SIM in positive (1.7 to 6 min) and negative (6.4 to 18 min) mode was acquired with R @ *m/z* 200 = 140,000; automated gain control of 5 × 10^4^; maximum injection time of 650 ms; isolation window of 20 Th; and scan range of *m/z* 400 to 1000 in positive and *m/z* 350 to 1200 in negative mode, respectively. The inclusion list of masses targeted in t-SIM analyses started at *m/z* 355 in negative and *m/z* 405 in positive ion mode, and other masses were computed by adding 10 Th increment (i.e., *m/z* 355, 365, 375, …) up to *m/z* 1005 in positive mode and up to *m/z* 1205 in negative mode. All acquired spectra were filtered by PeakStrainer (https://git.mpi-cbg.de/labShevchenko/PeakStrainer/wikis/home (accessed on 18 June 2020)) [50] and stitched together by an in-house-developed script [51]. Lipids were identified by LipidXplorer software [52]. Molecular fragmentation query language (MFQL) queries were compiled for ergosterol, ergosterol esters, PC, LPC, PE, LPE, PI, LPI, PA, LPA, PG, LPG, PS, LPS, TG, and DG lipid classes. The identification relied on accurately determined intact lipid masses (mass accuracy better than 5 ppm) and a signal-to-noise threshold higher than 3. Lipids were quantified by comparing the isotopically corrected abundances of their molecular ions with the abundances of internal standards of the same lipid class. Ergosterol, as well as ergosterol esters, were normalised to the internal cholesterol and internal CE standard, respectively.

### 2.7. Lipid Extraction and Quantification by Thin-Layer Chromatography

Yeast cultures were inoculated from stationary overnight cultures in SCD to fresh SCD medium to an OD600 of 0.1. Expression of *POR1* or *PSD1* was induced by shifting to SCD/SCG mixtures as described in the paragraph on growth conditions. In total, 80 OD600 units were harvested at 24 h after inoculation for experiments without additional overexpression, or at 24 h after shift for overexpression experiments. Total lipids were extracted with chloroform/methanol 2:1 (*v*/*v*) according to Folch et al. [53]. The organic phase was dried under a stream of nitrogen and dissolved in 500 µL of chloroform/methanol (2:1, *v*/*v*). A total of 30 µL of lipid extracts were sprayed on TLC plates using a CAMAG Linomat 5. PE (850757P), PS (840034P), and PC (850457P) were purchased from Sigma Aldrich and included as standards on each TLC plate. The PE standard was applied at 6 different dilutions to allow for calculation of a standard curve and determine absolute PE levels.

Neutral lipid separation and analysis was performed by thin-layer chromatography (TLC) on silica gel 60 plates (Merck), essentially as described before [54], using CHCl_3_/MeOH/water (32.5:12.5:2) mixture as mobile phase [55,56]. TLC plates were derivatized by dipping into 3.2% H_2_SO_4_ and 0.5% MnCl_2_, followed by carbonization at 120 °C for 30 min. Developed TLC plates were imaged using a Bio-Rad Universal Hood II, and bands were quantified using image J.

### 2.8. Statistical Analysis

**Figure 2:**
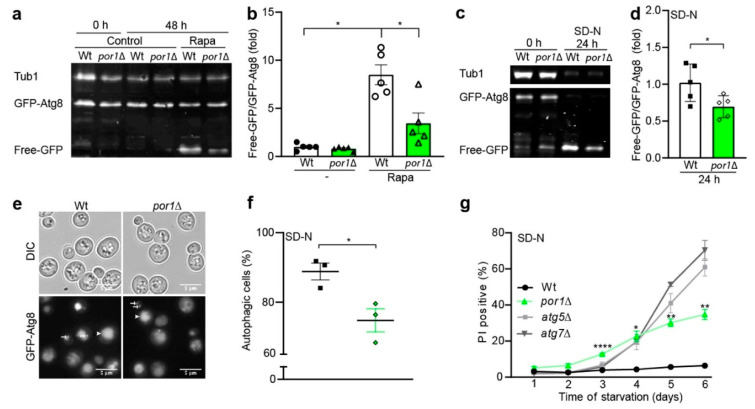
*por1*Δ cells showed reduced autophagic capacity upon rapamycin treatment or nitrogen starvation. (**a**,**b**) *POR1* deletion reduces the autophagic capacity at 48 h on SD medium upon rapamycin treatment, as shown by GFP–Atg8 liberation. A representative immunoblot is depicted in (**a**) and free-GFP/GFP–Atg8 ratios are quantified in (**b**). The immunoblots were probed with GFP- and tubulin-specific antibodies. Specific signals are normalised to wildtype (*n* = 5). (**c**–**f**) Autophagy induction by nitrogen starvation is reduced in *por1*∆ at 24 h. A representative immunoblot of wildtype (Wt) and *por1*Δ cells before (0 h) and after shift to nitrogen starvation media (24 h) is depicted in (**c**) and free-GFP/GFP–Atg8 ratios are quantified in (**d**). Immunoblot signals are normalised to wildtype (*n* = 5). Representative micrographs of GFP–Atg8-expressing wildtype and *por1*Δ cells are depicted in (**e**) and GFP–Atg8-positive vacuoles (autophagic cells) are quantified in (**f**) (*n* = 3, with *n* > 279 quantified cells per biological replicate). (**g**) *POR1* deletion increases levels of cell death on nitrogen starvation, as assessed by PI staining and flow cytometry (*n* = 4). PI-positive fractions of wildtype (Wt), *por1*Δ, *atg5*Δ, *and atg7*Δ cells are plotted in line diagrams showing mean ΔSEM. Statistical analysis was performed with GraphPad Prism using Welch’s ANOVA in (**b**), Welch’s *t*-test in (**d**), unpaired *t*-test in (**f**), and 2-way ANOVA in (**g**). Error bars indicate standard error of the mean (SEM) and asterisks in the figures indicate significant differences, * *p* < 0.05, ** *p* < 0.01, **** *p* < 0.0001.

Statistical analysis was calculated in GraphPad Prism8. To disprove the null hypothesis (no difference between conditions) a one-way ANOVA corrected with a Tukey *post hoc* test or Welch’s ANOVA (unequal variances) with Dunett’s post hoc test was performed. A two-way ANOVA analysis was performed in Figure 2g. A Student’s *t*-test was applied when comparing two groups only. Information on individual statistical tests is given in the figure legends. Error bars indicate standard error of the mean (SEM) and asterisks in the figures indicate significant differences, * *p* < 0.05, ** *p* < 0.01, *** *p* < 0.001, **** *p* < 0.0001. Figures were prepared with GraphPad Prism and Adobe Illustrator CS6 (Adobe). Microscopic pictures were processed with Fiji [57].

## 3. Results

### 3.1. Loss of POR1 Reduced Cellular Autophagy Levels

Por1 is an important regulator of mitochondrial and cellular homeostasis. Its deletion is associated with alteration of mitochondrial morphology and function [58,59,60]. *POR1* deletion only mildly affects growth on fermentable carbon sources, whereas growth on non-fermentable carbon sources is largely affected by *POR1* deletion (Figure S1a,b) [61]. Respiration-deficient Rho°-yeast cells have been shown to have reduced autophagy levels [62]. However, it is unknown whether *POR1* function is essential for autophagy. We, thus, conducted autophagy measurements using a *POR1* deletion mutant. We made use of the GFP–Atg8 liberation assay as a measure of autophagic flux [42,63,64]. GFP–Atg8 expression was achieved by N-terminal GFP tagging at its chromosomal locus. We detected liberation of free GFP from GFP–Atg8 in yeast cultures grown on YPD media by immunoblotting, which revealed a decrease in basic autophagy levels in *por1*∆ at both 24 and 48 h after inoculation (Figure 1a). We quantified the ratios of free GFP to GFP–Atg8 bands, which confirmed a significant decrease in autophagic flux in *por1*∆ at both time points (Figure 1b). To corroborate our data, we applied the Pho8∆60 assay as a second approach. This assay measures the autophagic flux by determination of alkaline phosphatase (ALP) activity of a truncated Pho8 phosphatase, which localises to the cytosol instead of the vacuole. Truncated Pho8 only gets activated upon autophagic delivery to the vacuole. ALP activity is, hence, a quantitative measure for autophagic flux [44]. Importantly, measurement of ALP activity in *por1*∆ confirmed our results above in that autophagy was significantly reduced in *por1*∆ strains (Figure 1c).

Next, we complemented the chromosomal *POR1* deletion by *POR1* expression from a pESC plasmid with the galactose-inducible Gal10 promoter. Por1 expression was assessed and quantified by immunoblotting, confirming complete lack of Por1 in knock-outs and re-expression in the pESC constructs (Figure S1c,d). Indeed, *POR1* expression re-established autophagy in *por1*∆ cells quantified by GFP–Atg8 immunoblotting (Figure 1d,e) and measurement of ALP activity (Figure 1f). It is noteworthy that overexpression of Por1 in wildtype cells did not result in additional upregulation of mean autophagy levels suggesting that natural Por1 expression levels are optimised for efficient autophagy induction. Furthermore, *POR1* expression was sufficient to compensate for chromosomal *POR1* loss, thus re-establishing wildtype-like growth behaviour (Figure S1e) and partial restoration of cell size, which is decreased in *por1*∆ (Figure S1f). Additionally, we investigated if the decrease in autophagy led to a loss in survival. We determined membrane integrity loss by flow cytometric quantification of propidium iodide (PI) staining and observed a significant increase in the PI-positive fraction in *por1*∆ cells after 24 and 48 h as compared to wildtype cells (Figure 1g). Por1 overexpression significantly rescued this loss in survival after 48 h.

The final degradation of autophagosomes occurs through fusion with the vacuole in yeast. To test whether defects in autophagy could be due to vacuole dysfunction, we investigated whether *POR1* deletion was associated with changes in vacuole morphology and function. We used mutant strains expressing a c-terminal GFP fusion of Vph1 to visualise vacuole membranes. Fluorescence microscopic observation indeed showed significantly smaller vacuoles in *por1*∆ cells as compared to wildtype (Figure S1g–i), measured by mean diameter (Figure S1h). Vacuoles appeared rather fragmented or multi-lobular (Figure S1i). In addition, Vph1–GFP fluorescence intensity seemed more inhomogeneous in *por1*∆ cells, indicating a potential change in vacuole acidification, which is essential for degradation. Indeed, staining with quinacrine, a pH-dependent dye accumulating in acidic compartments, showed an inhomogeneous but overall decreased fluorescence signal in *por1*∆ cells compared to wildtype (Figure 1h,i). While some cells show a normal vacuole acidification, the changes in vacuole function and morphology could still contribute to the defective autophagy observed in *por1∆* (Figure 1h).

In summary, we show that *POR1* deletion reduces cellular autophagy levels and that re-expression from a plasmid is sufficient to compensate for chromosomal *POR1* loss. Our results suggest that wildtype Por1-levels are optimised for efficient growth, autophagy, and survival, whereas a further increase in porin does not deliver additional benefit.

### 3.2. POR1 Deficiency Reduced the Autophagic Capacity upon Induction with Rapamycin or Nitrogen Starvation

Autophagy can be induced by different means, including different forms of starvation, e.g., nitrogen or glucose starvation, or by treatment with autophagy-inducing drugs, such as rapamycin [65]. We, thus, wanted to know whether the capacity to induce autophagy was diminished in *por1*∆. We, therefore, assessed GFP liberation from GFP–Atg8 by immunoblotting and ALP activity upon rapamycin treatment. Indeed, our experiments confirmed our initial assumption that loss of *POR1* limits the autophagic capacity (Figure 2a,b and Figure S2a–c). While rapamycin treatment significantly induced autophagy in wildtype cells, this capacity is reduced in *por1*∆. The autophagic response was not completely diminished in the *POR1* deletion strain, but rather showed a slower and incomplete reaction to rapamycin treatment (Figure 2b and Figure S2b,c). As a second means of nonspecific autophagy induction, we used nitrogen starvation. Quantitative analysis of GFP liberation attested a significant reduction in nitrogen-starvation-induced autophagy level in *por1*∆ as compared to wildtype (Figure 2c,d). GFP–Atg8 fluorescence microscopy pictures showed a much weaker vacuolar GFP signal (indicated with arrowheads) in *por1*∆ (Figure 2e). Notably, GFP–Atg8 dots (highlighted by white arrows) represent autophagosomes and are still detected in *por1*∆. Quantification of GFP-positive vacuoles revealed a significant decrease in autophagic flux for *por1*∆ (Figure 2f). Since autophagy is crucial to survive in times of starvation, we conducted a time course experiment on nitrogen starvation media to test whether *por1*∆ shows reduced survival. Propidium iodide (PI) staining was used as a measure of cell death, which was significantly increased in *por1*∆, starting from day 3 of the starvation experiment (Figure 2g). A loss of autophagy, as seen in cells lacking key autophagy-related genes, such as *ATG5* or *ATG7*, strongly reduces starvation survival. In line with loss of *POR1* leading to a reduced autophagic capacity, we observed an increase in PI positivity when compared to wildtype. This was, however, less pronounced than observed in *atg5*∆ and *atg7*∆ cultures. Altogether, our results demonstrate that *POR1* deficiency is associated with impaired autophagy correlating with a loss of survival upon nitrogen starvation.

### 3.3. POR1 Deficiency Was Associated with Specific Changes in the Lipid Profile, Particularly Affecting Phospholipids

**Figure 3:**
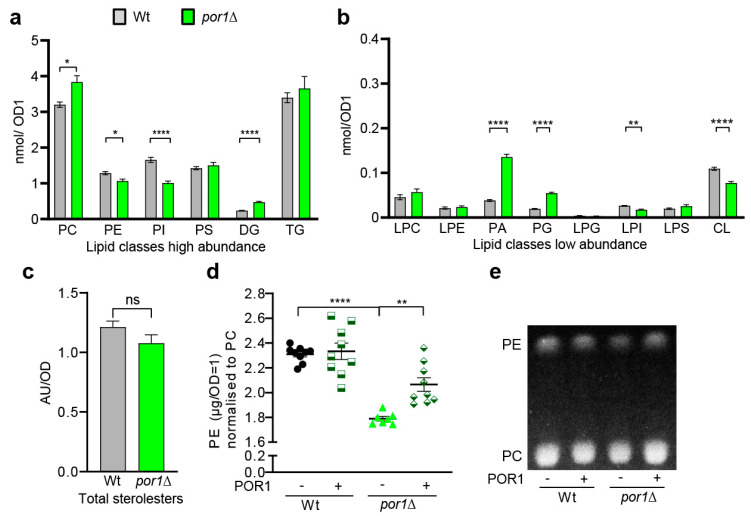
Porin deletion triggers lipidomic changes, including increase in PC and reduction in PE. (**a**–**c**) Total lipids of wildtype and *por1*Δ were detected by shotgun lipidomics. Lipid classes of high abundance (**a**), low abundance (**b**), and sterol esters (**c**) are shown in separate panels. All lipid classes were normalised to OD1 (*n* = 8). (**d**,**e**) Reduction in PE-to-PC ratios in *por1*∆ can be reverted by *POR1* re-expression. Quantification of PE-to-PC ratios based on TLC is shown in panel (**d**) (*n* ≥ 7) and a representative TLC section is shown in (**e**). Statistical analysis was performed with GraphPad Prism. To analyse the lipid profile changes selected, paired Welch’s ANOVA for high (**a**) and low (**b**) lipid classes was used. Unpaired *t*-test was used for (**c**) and Welch’s ANOVA for (**d**). Error bars indicate standard error of the mean (SEM) and asterisks in the figures indicate significant differences, * *p* < 0.05, ** *p* < 0.01, **** *p* < 0.0001.

Autophagy depends on autophagosomal membrane biogenesis, which relies on the availability of phospholipids. To investigate whether Por1 may play a role in the management of phospholipid supply, we conducted a lipidomic analysis using shotgun lipidomics. The lipid profile of *por1*∆ cells showed an increase in PC, DG, PA, and PG, whereas PE, PI, LPI, and CL abundance was reduced (Figure 3a,b). The neutral lipid TG (Figure 3a) and sterol esters (SE) (Figure 3c) were unaffected. The changes in mitochondria-specific lipids, with CL being reduced but its precursors DG, PA, and PG being increased, suggest that mitochondrial CL synthesis is affected in *por1*∆. This might reflect a mitochondrial state where limited mitochondrial function is possible, even when Por1 function is absent. The most prominent increase was detected for PA, which is largely upregulated in *por1*∆. PA has a key role as an intermediate between neutral lipid and phospholipid metabolism. Furthermore, it is a precursor for the mitochondrially produced lipids CL and PG. Increasing total PA levels might, thus, be an adaptive response to overcome reduced mitochondrial PA import. Of note, high PA levels maintain the transcriptional repressor Opi1 at the ER, which facilitates transcription of Opi1-regulated *INO* genes [66]. Since autophagosomal membranes mainly consist of PC, PE, and PI with a particularly high level of unsaturation [13], we further analysed the lipid species distribution of these lipid classes (Figure S3a–c). For PC, we noted a decrease for 32:2 and 34:2 species, whereas the corresponding species containing only one unsaturated FA were increased to the same extent. PE and PI species showed a shift towards increased incorporation of longer FA as compared to wildtype (Figure S3a–c).

As cellular PE has been identified as a major contributor to autophagy induction previously [17], we analysed total PE levels of *por1*∆ (Figure 3d) using thin-layer chromatography (TLC) (Figure 3d,e). Corroborating the lipidomic analyses, we were able to raise the total PE levels by re-expression of *POR1* from a plasmid (Figure 3d,e). With these changes in total PE and PE species distribution in *por1*∆ (Figure S3b), we reasoned that decreased PE synthesis in *por1*∆ could be causally linked to reduced autophagy.

### 3.4. PSD1 Overexpression Could Partially Compensate for the Loss of POR1

**Figure 4:**
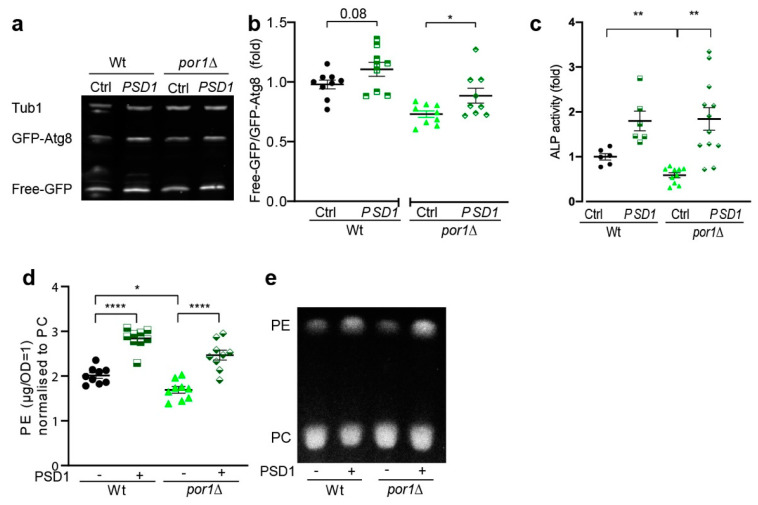
*PSD1* overexpression could partially compensate for the loss of *POR1*. (**a**–**c**) *PSD1* overexpression increases autophagy in *por1*Δ. A representative immunoblot is depicted in (**a**) and free-GFP/GFP–Atg8 ratios of wildtype (Wt) and *por1*Δ cells 24 h after induction of *PSD1* overexpression are plotted in (**b**). Immunoblots were probed with GFP- and tubulin-specific antibodies and the signals were normalised to wildtype expressing an empty vector (Ctrl) (*n* = 9). Alkaline phosphatase activity was assessed using the Pho8∆60 assay as a second readout to measure autophagic flux (*n* ≥ 6) (**c**). (**d**,**e**) *PSD1* overexpression increases PE-to-PC ratios in wildtype (Wt) and *por1*∆. Quantification of PE-to-PC ratios based on TLC is shown in (**d**) (*n* = 9) and a representative TLC section is shown in (**e**). Statistical analysis was performed with GraphPad Prism. Statistical analysis in (**b**) was performed by unpaired *t*-test, in (**c**) by Welch’s ANOVA, and (**d**) by one-way ANOVA. Error bars indicate standard error of the mean (SEM) and asterisks in the figures indicate significant differences, * *p* < 0.05, ** *p* < 0.01, **** *p* < 0.0001.

Phosphatidylserine decarboxylase 1 (Psd1) catalyses the decarboxylation of PS to form PE. Psd1 is dually targeted to mitochondria and the ER and, thus, can produce PE within both organelles depending on metabolic needs [21]. Especially since autophagosomal phospholipids have a particular need for unsaturated fatty acids, which might require de novo synthesis, we wondered whether inefficient targeting/activation of Psd1 could explain autophagy reduction in *por1*∆. We, thus, tested whether the autophagic capacity of *por1*∆ could be enhanced by *PSD1* overexpression. Psd1 overexpression indeed significantly increased autophagy in *por1*∆, as documented by GFP–Atg8 liberation and Pho8 assays (Figure 4a–c). Successful PE elevation by Psd1 overexpression was confirmed by TLC (Figure 4d,e and Figure S4a). Psd1 undergoes c-terminal cleavage upon import into the mitochondria, which represents a crucial step for its activation [20]. If mitochondrial uptake of Psd1 was inefficient in *por1*∆, decreased Psd1 processing should be visible on immunoblots as a size shift. We, thus, assessed relative abundance of the immunoblot bands corresponding to processed Psd1 upon *PSD1* overexpression. In line with our hypothesis, we found that Psd1 processing was impaired in *por1*∆ (Figure S4b,c). We conclude that *POR1* deletion affects cellular lipid homeostasis, which includes changes in PE content and species distribution. *PSD1* overexpression can partially restore autophagy in *por1*∆, which suggests that PE limitation in *por1*∆ is linked to autophagy impairment.

## 4. Discussion

VDACs are important proteins of the outer mitochondrial membrane with high abundance and involvement in multiple functions, which include metabolite shuttling [29,67], mitochondrial protein import [68,69], and formation of membrane contacts [70]. Mammalian VDACs have further been linked to carcinogenesis [71,72], which highlights their importance and focused interest as a current research subject. Here, we show that the yeast VDAC Por1 controls lipid homeostasis and suggest a causal link to the regulation of autophagy.

*POR1* deletion decreased basic autophagy levels and reduced the susceptibility to autophagy induction by rapamycin and nitrogen starvation. *POR1* deletion further triggered vacuole dysfunction, which could causally link to autophagy impairment in *por1*∆, as vacuole function is absolutely required for autophagy. Vacuole dysfunction itself could be a direct cause of altered lipid metabolism, or due to changes in signalling. The yeast AMPK orthologue Snf1 kinase, for example, has been connected to porins in yeast [73]. Another possible signalling candidate is the MAPKinase Slt2, which has been linked to vacuole function [74]. Whether or not these signalling routes are involved in Por1-regulated vacuole function and autophagy needs to be addressed in future experiments.

Our lipidomic analysis revealed significant changes in key phospholipids when comparing *por1*∆ and wildtype (Figure 3 and Figure S3). In fact, the three CL precursors DG, PA, and PG are highly upregulated in *por1*∆ (Figure 3b), which might represent a compensation pattern for the lack of CL. The fact that DG levels are not as highly increased, as compared to the relative increase in PA and PG, suggests that mitochondrial CL synthesis occurs predominantly through the Ups2–Mdm35 independent route, consuming DG under these conditions [18]. Thus, PA and PG are upregulated, but accumulate as they are not consumed for mitochondrial CL synthesis.

Our analysis further showed that *POR1* deficiency reduces total PE levels (Figure 3). A reduction in cellular PE might, thus, be causally linked to the decreased autophagic capacity as measured for *por1*∆ (Figure 1 and Figure 2). We reasoned that Por1 could either function in PS transfer from ER to mitochondria itself or be required for mitochondrial import of Psd1 or the Ups machinery required for phospholipid import through the inner mitochondrial membrane. Interestingly, Por1 has recently been reported to interact with Mdm35 and to be implicated in mitochondrial lipid homeostasis [58,60]. Two other recent publications further suggest a role for porin in mitochondrial import, which represents another possible line of explanation for its effects on autophagy [68,69]. A study in acute myeloid leukaemia cells suggests that mitochondria can control autophagy through the regulation of VDAC-involving mitochondria–endoplasmic reticulum contact sites (MERCs) to fine-tune lipid degradation [72].

Interestingly, Acc1, as well as Fas1, have been identified as crucial players in extending the autophagosomal membrane by de novo phospholipid synthesis during autophagy [13,14]. Schütter et al. suggest a new model of autophagosomal membrane extension involving localisation of Fas1 to the phagophore assembly site (PAS), which, in turn, drives FA synthesis and channels them into the ER. Increased FA abundance in the ER then triggers phospholipid synthesis, which are channelled back to the PAS via the Atg2/Atg18 complex to fuel autophagosomal membrane growth [13].

Mitochondria and the ER exchange PS and PA via membrane contact sites [26,28,75]. There is no evidence, so far, that PE is also channelled back from mitochondria to the ER. Recent advances suggest that PE might be directly synthesised in the ER, which is achieved by Psd1 translocation to the ER [21]. Autophagosomes are mainly composed of PC (38%), PI (37%), and PE (19%) [13]. The novel mechanism that autophagosomal membrane growth relies on de novo phospholipid synthesis in the ER requires PE supply by either Psd1 activity or via the ethanolamine-dependent salvage pathway (Kennedy pathway). *PSD1* overexpression has been shown to increase the autophagic capacity [17], whereas knockout of *PSD1* and *PSD2* resulted in reduced autophagy [76]. *PSD1* and *PSD2* single mutants have been studied regarding their role in mitophagy, as discussed later [77]. Since Psd1 accounts for 70–80% of total PSD activity in yeast [22], it can be assumed to be of predominant importance for autophagy. Still, the question remains whether and how the different pathways of PE synthesis offer redundancy regarding their role in autophagy and if they can compensate for each other. A Psd1-dependent raise in PE at the ER could, in principle, be achieved in two ways: either (i) producing PE in the mitochondria and then channelling it to the ER, or (ii) by targeting Psd1 itself to the ER. Friedmann et al. have shown that a subfraction of Psd1 indeed localises to the ER upon PE starvation [21]. Induction of autophagy by rapamycin treatment or nitrogen starvation is likely to induce such PE starvation conditions, triggering ER targeting of Psd1. Since *POR1* deletion has been associated with reduced efficiency in mitochondrial protein import [68,69], Psd1 uptake into mitochondria might be reduced as well. This hypothesis is supported by our anti-Psd1 immunoblots, suggesting decreased proteolytic activation of Psd1 (Figure S4b,c) in *por1*∆. Another recent study investigated the involvement of Psd1 during mitophagy in yeast [77]. Interestingly, the study reports that Psd1 is predominantly involved in nitrogen-starvation-induced mitophagy, whereas Psd2 is required for mitophagy induction in the stationary phase. The authors further suggest that defective mitophagy in *psd1*∆ is due to impaired recruitment of Atg8 to mitochondria. Even though we did not investigate specific forms of autophagy, such as mitophagy, here, our findings of porin 1 involvement in autophagy and its relation to Psd1 function are compatible with the results from Vigié et al.

For future research, it remains to be investigated whether porin function is needed for the extension of autophagosomal membranes or, rather, at the step of autophagy initiation/PAS formation.

## Data Availability

The data presented in this study are available in this article and supplementary material. Additional original data requests may be addressed to the corresponding authors.

## Supplementary Materials

The following are available online at https://www.mdpi.com/article/10.3390/cells10092416/s1, Figure S1: Additional growth and expression data regarding POR1 deletion and re-expression. Figure S2: Rapamycin mediated autophagy was impaired in por1Δ cells. Figure S3: Phospholipid species and total PE were significantly changed in por1Δ cells. Figure S4: PSD1 overexpression increased PE levels in wildtype and por1Δ cells.
