# Supplementary material for: Porin 1 Modulates Autophagy in Yeast
Source: Cells. 2021 Sep 14;10(9):2416. doi: 10.3390/cells10092416 (PMC8464718; doi:10.3390/cells10092416)
Supplement: Supplementary file 1 [file cells-10-02416-s001.zip › cells-1368995-supplementary.pdf]

Supplemental figures

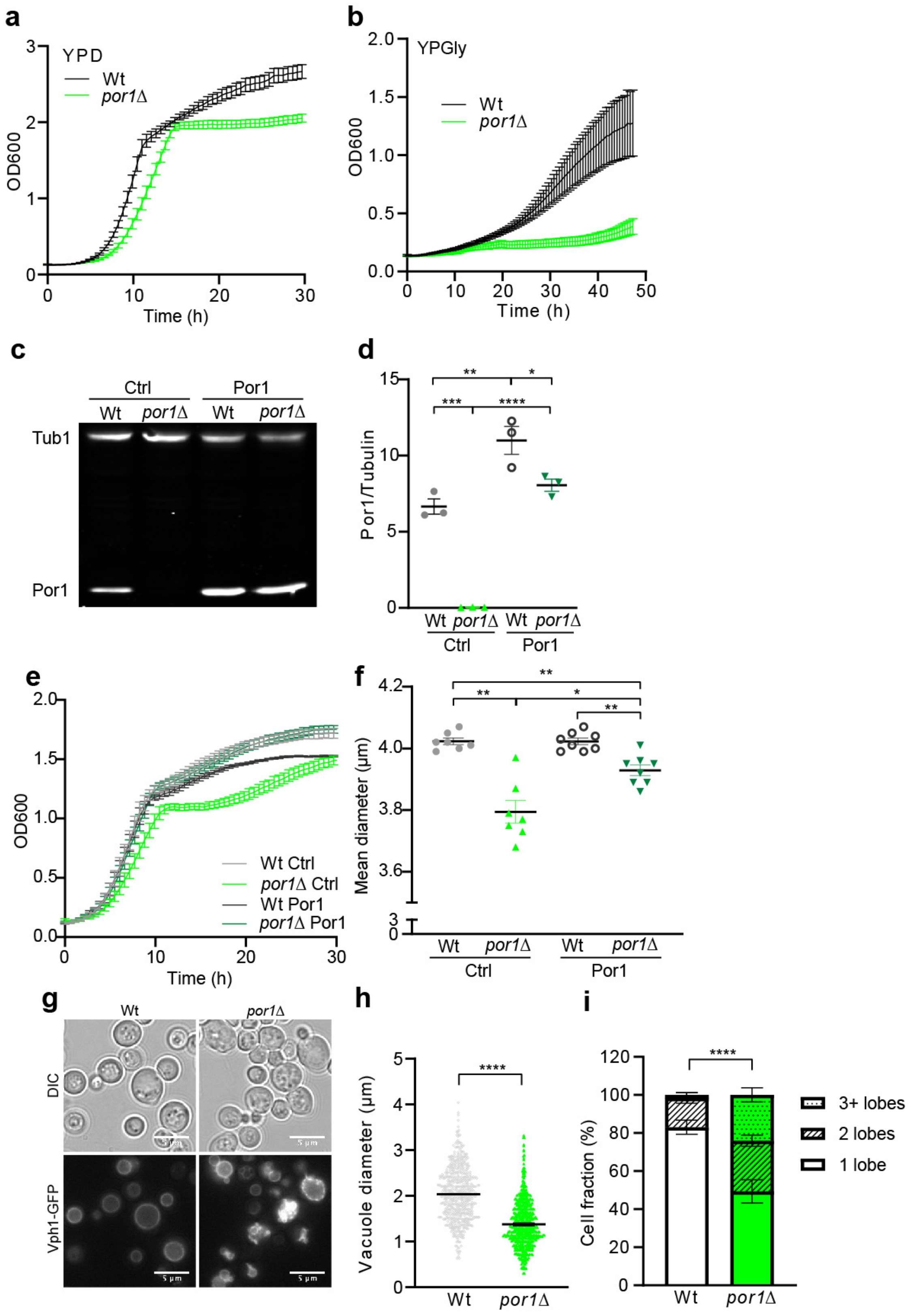

Figure S1: Additional growth and expression data regarding *POR1* deletion and re-expression. **(a, b)** *POR1* deletion decreases growth on YPD **(a)** which is exacerbated in YPGly **(b)**. OD_{600} was measured every 30 minutes with a SPECTROstar^{Nano} spectrophotometer (Means±SEM n≥14). **(c, d)** Por1 expression levels in wildtype (Wt) and *por1Δ* cells with or without *POR1*-expression were detected by

immunoblotting using specific Por1-antibodies. A representative immunoblot is shown in **(c)** and quantification of Por1/Tubulin ratios (n=3) is plotted in **(d)**. **(e)** The growth defect of *por1*Δ is restored by Por1 expression on SCD/SCG (1:1) media. OD_{600} was measured every 30 minutes with a SPECTROstar^{Nano} spectrophotometer (n=8) **(f)** The mean cell diameter is reduced in *por1*Δ cells as compared to wildtype (Wt), which can be partially restored by Por1-expression. Cell diameters were determined using a CASY cell counter (n≥7). **(g, h, i)** Microscopy of Vph1-GFP-expressing wildtype and *por1Δ* cells revealed that the vacuole diameter was reduced and the number of vacuoles per cell was increased in *por1*Δ. Representative microscopy pictures are shown in **(g)**. The vacuole diameters and numbers of vacuoles per cell are quantified in **(h)** and **(i)**, respectively. Micrographs were taken 24 hours after inoculation. Statistical analysis was performed with GraphPad Prism. Line graphs show means±SEM (error bars). Dot plots show data points along with the means (line) ±SEM. Welch-ANOVA was used in (d) and (f), Student T-Test in (h) and (i).

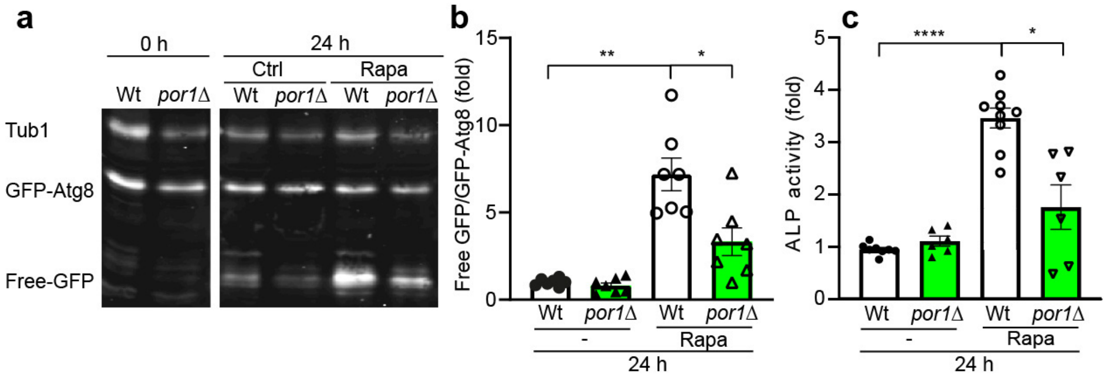

Figure S2: Rapamycin mediated autophagy was impaired in *por1*Δ cells. (a, b, c) A representative immunoblot of wildtype and *por1*Δ cells at 24 hours after treatment with 30 nM rapamycin is depicted in **(a)** and quantification of free-GFP/GFP-Atg8-ratios is shown in **(b)**. Blots were probed with GFP- and tubulin-specific antibodies and signals were normalised to wildtype (n=7). **(c)** Alkaline phosphatase activity was assessed at 24h using the Pho8Δ60 assay indicative for autophagic flux (n>7). Statistical analysis was performed with GraphPad Prism. Significance in (b) and (c) was assessed using Welch ANOVA.

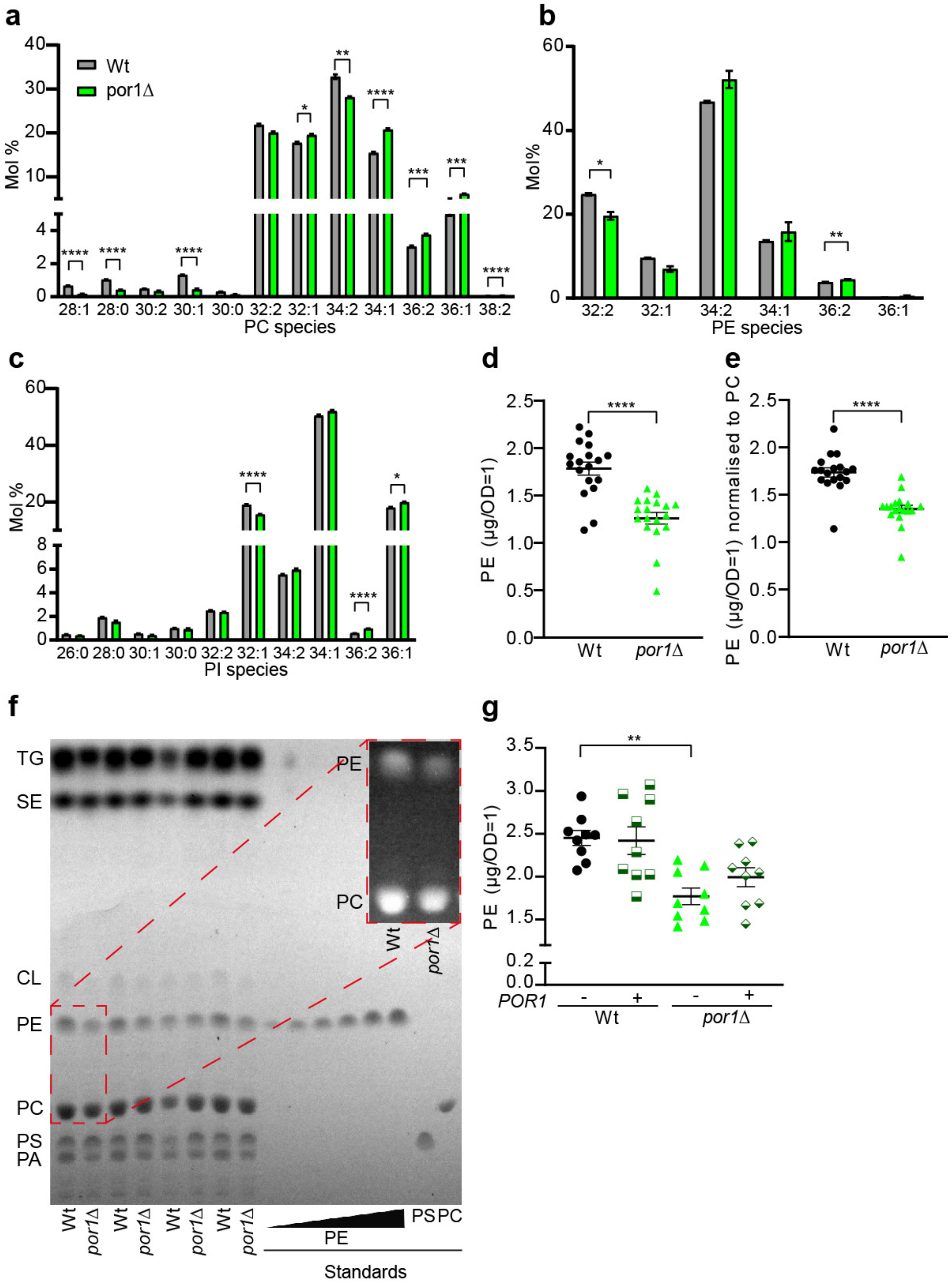

Figure S3: Phospholipid species and total PE were significantly changed in *por1*Δ cells. (**a, b, c)** Lipid species analysis of wildtype and *por1*Δ cells are shown for PC **(a)**, PE **(b)** and PI **(c)**. All lipid species are shown in mol % (n=8). **(d, e, f)** TLC analysis for quantification of PE. Absolute PE levels are shown in **(d)** and the same data normalised to PC are shown in **(e)**. A representative TLC plate is shown in **(f)** with an inverted magnification of PE and PC bands from the first two lanes in the red rectangle. **(g)** The absolute PE content with *POR1* re-expression from a plasmid is quantified in (g). Statistical analysis was performed with GraphPad Prism. Welch ANOVA was used as statistical analysis for (a) (NoC 276), (b) (66 NoC) and (c) (NoC 190). NoC: Number of Comparisons. Statistical significance in (d) and (e) was assessed using student t-test and in (g) by ordinary one-way ANOVA.

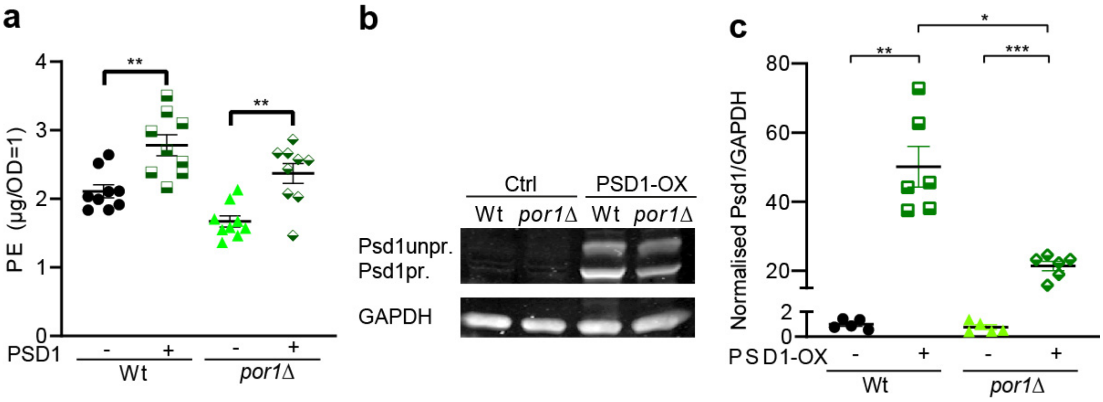

Figure S4: *PSD1* overexpression increased PE levels in wildtype and *por1*Δ cells. (a) Absolute PE levels were quantified by TLC. (**b, c)** Psd1 expression levels were detected by immunoblotting. A representative immunoblot monitoring *PSD1*-overexpression-levels at 24 hours after induction is shown in (b). Blots were probed with Psd1- and GAPDH-specific antibodies and signals of Psd1/GAPDH were normalised to wildtype cells expressing the empty vector (n=5). After mitochondrial import unprocessed Psd1 (Psd1unpr.) is cleaved into its processed, active form (Psd1pr) resulting in a size shift. Statistical analysis in (a) and (c) was performed with GraphPad Prism using one-way ANOVA.
